# The multiplex PCR assay detection of *Staphylococcus sciuri* antibiotic resistance, *mecA* gene, and the inhibitory effect of root exudate of *Nigella sativa* (black seeds) treated with magnetized water

**DOI:** 10.25122/jml-2021-0280

**Published:** 2022-02

**Authors:** Anas Al-Hayawi

**Affiliations:** 1. Biology Department, College of Education for Pure Sciences, Tikrit University, Tikrit, Iraq

**Keywords:** Multiplex PCR, *mecA*, root exudate, *S. sciuri*, *Nigella sativa*, magnetized water

## Abstract

121 bacterial samples isolated from wounds from both sexes and all age groups were collected from Salahadin General Hospital, Salahadin provenance, Iraq. Only 8 *Staphylococcus* *sciuri* (*S. sciuri*) isolates were identified. The bacterial isolation showed the highest sensitivity to Amoxicillin/Clavulanic acid, Cefotaxime, Methicillin, Streptomycin, and Vancomycin and resistance to all other antibiotics. The root exudates of black seeds were used for 10 and 20 days for both treatments with and without magnetized water, and the exudates were superior when using magnetized water for 20 days. Antibiotic resistance and the *mecA* gene were investigated, and a multiplex PCR assay was used to detect the *mecA* gene in *S. sciuri*. Optimized conditions were used to amplify *mecA* fragments that encode methicillin resistance.

## Introduction

*Nigella sativa* (the black seed) is part of the *Ranunculaceae* family, and it is widely cultivated in Iraq in some regions of the center and north. It is considered one of the most important and famous medicinal plants globally, with many benefits. The seeds are disinfectant and used as anti-intestinal parasites remedies, especially in children. Moreover, these seeds are helpful for asthma, strengthening the immune system or during the menstrual cycle [1]. In addition, the black seed is used in many sweetened foods as a remedy for stomach ailments and diuretics. It is widely cultivated on many soils and under different types of irrigation [2].

The magnetic field affects the angle of hydrogen binding to oxygen in the water molecule, as it decreases from 104° to 103°, forming cluster groups of 6–7 molecules compared to 10–12 molecules in the normal state. The small groups of water molecules formed from exposure to a magnetic field absorb the best mineral elements from the plants’ roots and enter faster through the root hairs [3]. Magnetized water changes its properties, becomes more streamlined, has high polar efficiency, the speed of vibrations of its molecules, and its absorption of ions increases, which leads to the rapid dissolution of crystals, causing the plant to absorb a greater number of salts faster [4].

The roots have many functions, such as releasing secretions, which are complex mixtures of organic and inorganic compounds secreted into the soil and limiting phagocyte interference below the soil surface [5]. The latter is determined by multiple factors, including the plant species, the stage of plant growth, the type of living organisms, and the different environmental factors [6]. The roots excrete water, oxygen, and inorganic ions, mainly composed of carbon, estimated 5–60% of the total carbon [7].

*Staphylococcus sciuri* (*S. sciuri*) is one of the pathogens found on the skin and the mucous membranes of animals, especially pets, and in farms with cows and sheep, as well as with wild animals [8]. This pathogen can also be found in foods that contain meat [9]; it abounds in environmental places with long-term storage such as water tanks and soil [10]. It can be isolated from the pharynx and nose and the reproductive and urinary systems in humans [11]. In recent times, there has been increased interest in detecting this bacterium due to negative consequences, especially the increasing spread in the hospital environment and involvement in urinary tract infections (UTIs), peritonitis, endophthalmitis, and wound infections [12, 13]. It is noteworthy that antibiotics could be discharged into the wastewater because many households get rid of the antibiotics by throwing them in the bathroom. A noticeable concentration of the antibiotics remains in the urine of the patients [14, 15]. However, many treatment methods could be used to remove the antibiotics from water/wastewater, such as electrocoagulation [16–19], electrolyzing [20–25], application of some types of adsorbents [26–28], chemical or natural coagulants [29–32] and combined methods [33–35]. Additionally, monitoring antibiotics in water/wastewater can be achieved using remote technologies, such as sensor technology [36–38] that enjoy a wide range of applications in many industries, such as monitoring chemicals [39, 40], properties of materials [41–43], and communications [44].

This study aimed to identify the prevalence of *S. sciuri* in Salahadin General Hospital, Iraq, and investigate the effects of magnetized water used in extracting black seed root exudates in reducing the antibiotic resistance of bacteria for the *mecA* gene. Finally, this study aimed to assess the effect of exudates in reducing the *mecA* gene expression of the plasmids.

## Materials and Methods

### Black seeds samples and root exudates

The black seeds were collected from the College of Agriculture, University of Baghdad, and were diagnosed and classified by the herbarium of the Natural History Museum of the University of Baghdad. Root exudates from black seeds were collected using the Hydroponic Culture System (HCS). The seeds were sterilized for two minutes with 1% NaClO, washed with sterile distilled water, covered with two layers of sterile gauze, and placed in a sterile 500 ml glass vial filled with sterile distilled water. The seeds were left to germinate, and the filtrate was collected after 10 and 20 days [45]. Following this, the root exudates were filtered with a Millipore filter of 0.22 μm, stored at 4°C until used on a Muller Hinton Agar. A freshly isolated inoculum was spread, two wells of 6 mm diameter were prepared using a sterile cork borer, and 50L of root exudates were filled into the wells and incubated at 37°C for 18–24 hours [46].

### Magnetized water

A dipole magnet (Magnetotron) was used by the laboratories of the Ministry of Science and Technology, Iraq. The strength of the magnet was confirmed by measuring the magnetization intensity with a Teslameter (F.W. Bell/Gauss, Model 5070, USA). The water passed through a tube in the magnet that contained an electric coil in which an electric current passed at a voltage of 220 volts to generate a magnetic field of 1500 gauss. The magnetic system was sterilized daily, and the root filter was passed every day through the system to ensure the continuity of the magnetization of the root exudates.

### Sampling and diagnosis

121 samples were collected from wounds at Salahadin General Hospital during November and December 2020. Samples were taken from both sexes and all age groups. According to Whitman *et al.* [46], microscopic and biochemical diagnostic experiments were performed, and the diagnosis was verified using the VITEK 2 compact method (Biomerieux, France).

### Antimicrobial susceptibility test

Antimicrobial susceptibility of bacteria was performed on each of the isolates against 10 antibiotics, including Amoxicillin/Clavulanic acid, Cephalexin, Cefotaxime, Methicillin, Erythromycin, Gentamicin, Streptomycin, Rifampin, Tetracycline, and Vancomycin. The tests were performed using the Kirby-Bauer wells method, using 25 mg/ml as a stock solution and 50 μg/ml as final consternation. Antibiotic powders were obtained from the Samarra Drugs Industry (SDI), Iraq. Antibiotics solutions were used as stock solutions and were prepared by dissolving an amount of the antibiotic in 1 ml distilled water. All the stock solutions were sterilized with a Millipore filter (0.45 μm) and kept under 20°C until use.

### Molecular identification of the mecA gene

Geneaid^TM^ Midi Plasmid Kit (PI025) (Geneaid Biotech Ltd., Taiwan) was used to extract plasmid DNA from *S. sciuri*. Multiplex PCR was used to detect *mecA* gene resistance. The region was amplified by multiplex PCR primers *mecA* 1 (5’-AAA ATC GAT GGT AAA GGT TGG C-3’) and *mecA* 2 (5’-AGT TCT GCA GTA CCG GAT TTG C-3’ [47]. The Multiplex PCR PreMix Kit (Bioneer, Korea) was used for PCR (Bio-Rad) reactions following the manufacturer’s instructions. The conditions of PCR amplification were as follows: 94°C for 3 min; 94°C for 30s, 55°C for 30s, 72°C for 30s for 30 cycles; and a final extension at 72°C for 4 min.

## Results

121 samples were collected from patients, and 44 of these showed bacterial growth on the primary culture media. *S. sciuri* was present on 8 of 44 samples, while the remaining samples did not show any bacterial growth. The lack of bacterial growth may be due to variations in dosage, inadequate use of antibiotics, or diverse strains of bacteria.

**Figure 1.**
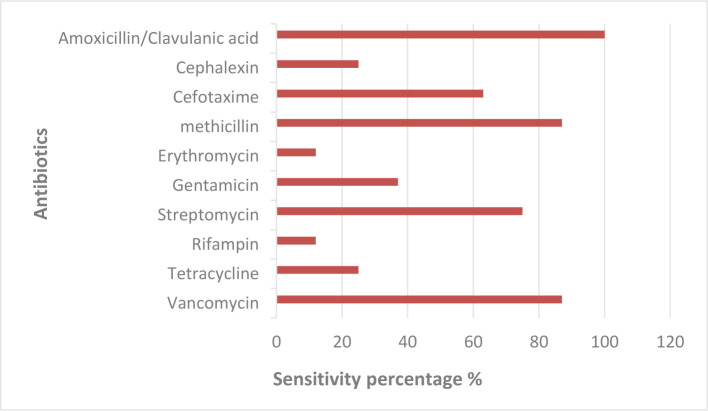
Antimicrobial Susceptibility test of 10 Antibiotics.

The primary purpose of testing the resistance of bacterial isolates to antibiotics is to identify the relationship between antibiotic resistance and the genetic content of bacteria isolated from patients. The drug susceptibility screening test was conducted on bacterial isolates using ten widely used antibiotics. Sensitivity and resistance were identified by observing the presence or absence of growth and measuring the diameter of the inhibition zone. The results of the studied isolates showed a difference in the levels of sensitivity and resistance to antibiotics (Figure 1). The bacterial isolates showed the highest sensitivity to Amoxicillin/Clavulanic acid, Cefotaxime, Methicillin, Streptomycin, and Vancomycin and were resistant to all other antibiotics. Amoxicillin is the first line of treatment for bacteria, and Clavulanic acid affects the inhibition of the Beta-Lactamase (BLs) enzyme and stops its action to prevent the degradation of the antibiotic, thus providing an opportunity for Amoxicillin to bind with the bacteria and stop its action [48]. The resistance of bacteria to antibiotics may be due to an inhibitory enzyme encoded by plasmids, or due to a transformation in the ribosomal unit, the 30S that is the center of the antibiotic’s effect, or to the lack of entry of the antibiotic into the bacterial cell [19].

The seeds were grown in sterile conditions using the Hydroponic Culture System (HCS), and the root exudates were collected in two different periods. The HCS were divided according to the quality of the water. The first group used sterile distilled water to grow the seeds. The second group used magnetized distilled water, and the exudates were collected in two periods, 10 and 20 days after inoculating the bacterial isolates on Mueller Hinton Agar medium and reading the inhibition diameter zone after 24 hours.

The results are shown in Table 1 after using root exudates of black seeds for a period of 10 and 20 days for both treatments with and without magnetized water. The diameter of the inhibition zone was 6 mm after treatment with black seeds only (ReBS) after 10 days, which is less than 8 mm after 20 days of treatment. Treatment with magnetized water after 10 days showed that the diameter of the inhibition zone increased by 14 mm compared to the treatment with black seeds only. When treatment time was increased to 20 days, there was a significant increase in the inhibition zone diameter (19 mm), a significant difference from treatment with black seeds alone.

**Table 1.** Diameter of inhibition zone of black seed roots exudates with and within magnetized water two times.

**Days**	**DW**	**Mw**	**ReBS (mm)**	**ReBS+Mw (mm)**
**10**	0	0	6	14
**20**	0	0	8	19

DW – Distilled water; Mw – Magnetized water; ReBS – Root exudates of black seeds; ReBS+Mw – Root.

The allelopathic effect of black seeds on both the root exudates with and without magnetized water, in most cases, inhibited bacterial growth. Active chemicals found in *Nigella sativa* are many phenolic derivatives and thymoquinone compounds which greatly help reduce bacterial infection and kill bacteria [49]. Bacteria can transport toxic molecules from inside the cell to the outside through efflux pumps. The compounds excreted in the black seeds root exudates may stop these pumps and precipitate the compounds excreted inside the cell without exposing them to the outside, thus reducing bacterial resistance and killing them [50].

The water we drink or use during our normal day is considered to lose many of its properties due to desalination processes or environmental pollution. This is also known as “dead water” due to its exposure to condensation and high air pressure during desalination and the addition of many sterile materials that lost many of their vital properties [51]. The magnetization of water restores the lost properties and reorganizes the charges of the water correctly when the shape of these charges is random in the current water. Furthermore, when water passes through the magnetic field, the hydrogen ions soluble heavy metals charge. This charge causes a temporary separation of the water molecules and then re-improves the taste of water [52]. The magnetized water reduces bacterial infections, as it gets its food through the cell membrane itself, as it absorbs a large amount of water through it. The magnetized water dissolves the cellular starch ions, leading to cell explosion (death) due to swelling [53]. The study conducted by Al-Hayawi and Al-Jubori [54] on the root exudates of onion and garlic on *E. coli* without magnetized water showed that an increase in the days of the root exudates led to an increase in the concentration of compounds excreted in the water. Moreover, the root exudates of garlic had a significant effect on the 14^th^ day of the study, and the time gave a higher antimicrobial effect by increasing the diameter of the inhibition zone.

**Figure 2.**
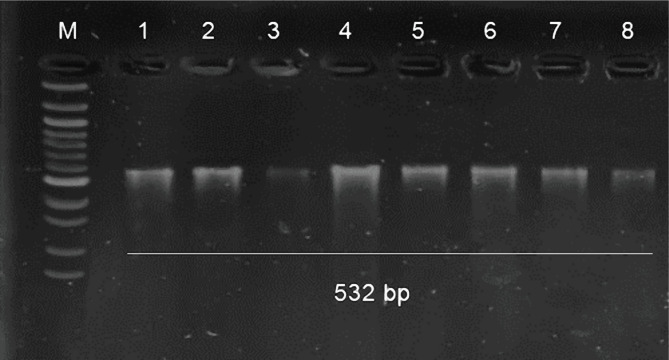
Electrogram of PCR product showing the band size (532 bp) of the mecA gene, the product was electrophoresed on 2% agarose at 5 volt/cm^2^, DNA ladder (100) lane M., lane 1–8. The sample refers to the S. sciuri mecA gene.

The presence of the *mecA* gene was extracted from plasmid DNA in 8 isolates of *S. sciuri*. The multiplex PCR assay was used to detect the *mecA* gene in *S. sciuri*, which produced visible bands (523 bp) on a 2% agarose gel (Figure 2).

The results showed that the *S. sciuri* isolates contain the *mecA* gene; the latter is responsible for resistance to the methicillin antibiotic, and this result agrees with previous studies on the same gene [55–57]. The multiplex PCR assay was used in this study for its ability to diagnose a gene or multiple genes in the same interaction and for the accuracy and speed of the results in the diagnosis of antibiotic resistance genes [58]. The *mecA* gene was selected based on clinical considerations, such as the association with *S. sciuri* resistance to clinically relevant antibiotics [59] and the prevalence at the Salahadin General Hospital. There is a great variation in the proportion of *Staphylococcus spp.* strains resistant to methicillin, as seen among different institutions. *Staphylococcus spp.* rates of antibiotic resistance range from 5% to over 50%, and central nervous system (CNS) resistance varies from 50% to 80% [60]. Vancomycin is routinely used to treat CNS infections despite documented oxacillin susceptibility due to this high prevalence and concern about undetected resistance. The *mecA* gene was also diagnosed because this gene is responsible for resistance to some antibiotics, such as methicillin. The results showed that the use of magnetized water with root exudates in two different periods led to a decrease in resistance rates among bacterial isolates that contain this gene.

## Conclusions

The present study focused on detecting *Staphylococcus sciuri* antibiotic resistance, the *mecA* gene, and the inhibitory effect of root exudate of *Nigella sativa* (black seeds) treated with magnetized water. Based on morphological characteristics and molecular analysis, the *mecA* gene was diagnosed using multiplex PCR assay detection, and black seed root exudates were treated with and without magnetized water in HCS compared to antibiotics.

Future studies should use sensor technology to monitor the antibiotic concentrations in patients’ blood. Additionally, the sensor technology can be used to monitor the concentration of antibiotics in the hospitals’ wastewater.

## Acknowledgments

### Conflict of interest

The author declares no conflict of interest.

### Ethical approval

The study was approved by the Ethics Committee of the Biology Department, College of Education for Pure Science, Tikrit University (approval no. S07/2326, 28.09.2020).

### Authorship

AY contributed to conceptualizing, methodology, original draft, manuscript editing, data collection, curation, and analysis.
